# Proceedings of the Buffalo Medical Association, Sept. 5, 1876

**Published:** 1876-10

**Authors:** 


### Proceedings of Medical Societies.

———

PROCEEDINGS OF THE BUFFALO MEDICAL ASSOCIATION, September 5th, 1876.

———

The President, Dr. Wyckoff, in the Chair.

Members present:—Drs. Gay, Coxe, Abbot, Bielby, Green, Burghart and Hebenstreit. By invitation, Drs. Baker and McCartey.

The Secretary being absent, on motion, Dr. McCartey was requested to act in that capacity, *pro tempore.*

DR. WYCKOFF being called away, DR. Gay was requested to take the chair.

DR. COXE was called on to read the essay of the evening, when it was moved by Dr. Bielby, and seconded by Dr. Abbott, that Dr. Coxe be requested to postpone the reading of his essay until the next meeting of the Association, when there would be a fuller house and consequently a larger appreciation of its merits. Carried.

*Reduction of dislocated Hip of two hundred and seventy days standing.*—Dr. Gay called Dr. Green to the chair, an reported a case of ancient dislocation of the femur of two hundred and seventy days standing. The patient was a man aged sixty, weighing 180 pounds. He reduced the dislocation and the patient has since done well. Will give a complete report of the case at some future meeting.

*Osteo-myelitis of humerus, amputation.*—Dr. Gay then presented some bones for the inspection of the members. One was the head of a humerus, with the upper third of the shaft.

The case, a woman 32 years old, suffering with osteo-myelitis of the left humerus, of syphilitic origin. There was an opening near the elbow, through which a probe could be introduced into the joint. He resected at the elbow, when he found that the medullary substance of the humerus was destroyed. He then amputated near the shoulder; pus escaped, and he could run his finger around in the center of the bone. He then, by Dr. McGraw's method, removed the head, ligating afterwards. The operation was performed three months ago. The patient now has a beautiful stump, with the rotundity of the shoulder preserved.

*Necrosis of tiba, exsection.*—The doctor also presented a tibia, that he had removed five weeks ago, from a child six years old.

The child had been injured some time previously, and afterwards an abcess formed, and when he examined it he found the tibia necrosed to its full extent, necessitating either amputation or exsection. He chose the latter operation, and at the hospital, in the presence of some of the students, excised the entire bone, with the exception of the upper epiphysis.

Recovery for a time seemed doubtful, but now the patient is doing well, and the doctor thinks the bone will be reproduced.

There was no splint used. The fibula gave all the support that was necessary.

*Epithelioma of vulva.*—Dr. Green reported a case of cancer of the Labia Majora. The patient was 43 years old, and was operated on by Drs. White, Brush and himself four months ago. The disease reappeared again and again, making it necessary to repeat the operation four times; at one time removing the clitoris and cutting down into the rectum. A part of the time the patient was in a bad state, always having pain, which was sometimes severe, and principally located in the gluteal region, until after the last operation, which was performed five weeks ago. Since then there has been no pain, the patient has improved in appearance, and her appetite has become good. The wound is not yet entirely healed, but looks well, has healthy granulations, and no sign of a return of the disease.

He thinks the operation will prove a success, and strongly advocates operative proceedure in such cases.

*Prevailing diseases.*—The doctor reported Cholera Infantum as prevailing in his practice; also Cholera Morbus, and some cases of Malarial fever.

On motion, the meeting adjourned.

